# Manifestations thromboemboliques chez 36 patients Ouest Africains infectés par le VIH

**DOI:** 10.11604/pamj.2018.31.224.13774

**Published:** 2018-12-06

**Authors:** Frédéric Nogbou Ello, Lidaw Déassoua Bawe, Gisèle Affoué Kouakou, Chrysostome Melaine Mossou, Doumbia Adama, Alain N'douba Kassi, Dine Mourtada, Eboi Ehui, Aristophane Tanon, Christophe Konin, François Eba Aoussi, Aka Rigobert Kakou, Serge Paul Eholié

**Affiliations:** 1 Service des Maladies Infectieuses et Tropicales, Centre Hospitalier Universitaire de Treichville, Abidjan, Côte d'Ivoire; 2 Institut de Cardiologie d'Abidjan, Côte d'Ivoire

**Keywords:** Maladie thromboembolique, VIH, Afrique de l´Ouest, Thromboembolic disease, HIV, West Africa

## Abstract

Chez les patients infectés par le VIH, la maladie thromboembolique est une complication dont le risque est accru. En Côte d'Ivoire, dans le service de référence de prise en charge médicale des personnes atteintes du VIH/SIDA, aucune étude n'a été menée sur la question. L'objectif de notre étude est de décrire les manifestations thromboemboliques colligées dans le Service des Maladies Infectieuses et Tropicales (SMIT) chez les patients infectés par le VIH, traités ou non par les antirétroviraux. Il s'est agi d'une étude rétrospective des dossiers de patients infectés par le VIH, hospitalisés, et présentant une thrombose veineuse profonde (TVP), artérielle et/ou une embolie pulmonaire de la période de janvier 2005 à juillet 2015. Le diagnostic a été posé par l'écho-Doppler des vaisseaux et/ou l'angioscanner thoracique. L'analyse a porté sur les aspects diagnostiques, thérapeutiques et évolutifs. Les dossiers de 36 patients dont 23 femmes (64%), sex-ratio H/F à 0,57, et âge moyen de 43±12 ans ont été retenus. Les thromboses veineuses profondes (TVP) ont été retrouvées chez 26 (72,2%) patients, des embolies pulmonaires (EP) chez neuf (25%) patients, une thrombose artérielle chez un patient (2,8%). La TVP était unilatérale dans 81% des cas et plus située à gauche (77%). L'EP était unilatérale et à droite dans 100% des cas et la thrombose artérielle était bilatérale dans 2,7% des cas. Chez les patients atteints de TVP, la veine fémorale (39%) et la veine poplitée (35%) étaient les sièges plus fréquents de thrombose. L'EP concernait les artères pulmonaires dans 77,8% des cas et la thrombose artérielle concernait les carotides internes gauche et droite. La majorité des patients était sous traitement antirétroviral (69%). Les infections opportunistes fréquemment associées étaient les candidoses orales (31%) et la tuberculose (33%). L'évolution a été marquée par neuf décès (25%). Cette étude rapporte une fréquence élevée des TVP au cours de l'infection à VIH. D'autres études s'avèrent nécessaires pour mieux appréhender le rôle du VIH dans la survenue de la maladie thromboembolique.

## Abstract (en)

In HIV-infected patients thromboembolic disease is a complication linked to heightened risk. In Ivory Coast no study has been conducted on HIV-infected patients treated in HIV Services. The aim of our study is to describe HIV-associated thromboembolic manifestations in patients treated or untreated with antiretroviral drugs whose data were collected in the Infectious and Tropical Diseases Service (ITDS). We conducted a retrospective study by reviewing the medical records of HIV-infected patients hospitalized with deep vein thrombosis (DVT), arterial thrombosis and/or pulmonary embolism over the period January 2005-July 2015. Diagnosis was based on Doppler ultrasound of vessels and/or on thoracic angioscanner. Diagnostic, therapeutic and evolutionary features of thromboembolic manifestations in these patients were analyzed. The medical records of 36 patients, including 23 women (64%), with a sex-ratio M/F of 0.57 and an average age of 43±12 years were selected. Deep venous thrombosis (DVT) was found in 26 (72.2%) patients, pulmonary embolism (PE) in 9 (25%) patients and arterial thrombosis in 1 patient (2.8%). DVT was unilateral in 81% of cases and predominantly left-sided in 77% of cases. PE was unilateral and right-sided in 100% of cases while arterial thrombosis was bilateral in 2.7% of cases. In patients with DVT, the femoral vein (39%) and the popliteal vein (35%) were most commonly affected by thrombosis. PE involved the pulmonary arteries in 77.8% of cases while arterial thrombosis involved the left and right internal carotid. The majority of patients was under antiretroviral treatment (69%). The most commonly associated opportunistic infections included oral candidiasis (31%) and tuberculosis (33%). Nine patients died (25%). This study highlights high rates of DVT in HIV-infected patients. Other studies are necessary to better understand the role of HIV in the occurrence of thromboembolic disease.

## Introduction

L'infection à VIH est connue comme un facteur associé des pathologies cardio-vasculaires [1-4]. La maladie thromboembolique (MTE) est retrouvée avec une incidence 2 à 10 fois plus élevée chez les patients infectés par le VIH comparée à la population générale [5]. En dehors de l'état prothrombotique dû au VIH, plusieurs mécanismes intriqués (le virus, le traitement antirétroviral, le vieillissement accéléré) ont émergé comme hypothèses de la maladie thromboembolique chez le sujet infecté par le VIH. On pourrait ajouter dans le contexte tropical, le déficit nutritionnel, et le paludisme, connus pour leur sévérité plus importante chez les Personnes Vivant avec le VIH (PVVIH) et leur effet délétère sur les vaisseaux sanguins [6-8]. Ces conditions contribuent à une vasculopathie et à l’hypercoagulabilité observées chez les PVVIH [9-12]. Bien plus, cet état d'hypercoagulabilité expose au risque de maladies thromboemboliques (MTE) sévères engageant le pronostic vital des PVVIH [13]. En Afrique de l'Ouest, on dispose de peu d'informations sur la MTE chez les PVVIH [14]. Devant l'augmentation croissante du nombre de patients exposés au cours des multi thérapies antirétrovirales, le vieillissement des populations suivies dans les cohortes et la survenue potentielle de maladies thromboemboliques, il nous est apparu important de rapporter les cas de MTE observés au Service des Maladies Infectieuses et Tropicales (SMIT) du Centre Hospitalier de Treichville à Abidjan, service de référence pour la prise en charge des adultes infectés par le VIH en Côte d'Ivoire.

## Méthodes

De janvier 2005 à décembre 2015, trente-six patients infectés par le VIH-1, hospitalisés au SMIT ont eu un diagnostic confirmé de MTE. Nous avons recueilli dans la base de données électroniques du service et à partir du dossier médical des patients, les données socio-démographiques, cliniques, biologiques et thérapeutiques (âge, sexe, statut VIH, facteurs de risque de thrombose (antécédent de thrombose veineuse ou d'embolie pulmonaire, chirurgie récente, alitement de toute origine), stade clinique, infections opportunistes, délai de diagnostic, symptômes de la thrombose, valeur des derniers lymphocytes CD4, traitement antirétroviral en cours). Nous avons défini comme manifestations thrombotiques, les thromboses touchant une artère ou une veine profonde. Le diagnostic de thrombose veineuse profonde (TVP) s'est fait par le Doppler artério-veineux (VividTM S6 de GE healthcare à deux dimensions avec option de contraste vasculaire de type « wide aperture»). Les embolies pulmonaires (EP) ont été confirmées par un angioscanner thoracique (Scanner à 64 barrettes de General Electrique, selon la technique d'acquisition spiralée volumique sur le thorax sans et avec injection de produit de contraste au temps artériel des coupes axiales avec un débit de 4 ml/secondes). La saisie et l'analyse statistique des données s'est faite sur le logiciel Epi Info version 7. Les variables quantitatives ont été décrites en termes de moyennes et d'écart-type. Les variables qualitatives ont été décrites en termes d'effectif et de pourcentage.

## Résultats

### Données générales

Parmi les 36 cas, nous avons dénombré 26 thromboses veineuses profondes, neuf embolies pulmonaires et une thrombose artérielle. L'ensemble des 36 patients (32 ivoiriens (89%), 3 burkinabés (8.3%), 1 malien (2,7%)) de l'étude était infecté par le VIH-1, majoritairement composé de femmes, avec un âge moyen de 43 ans, 69% d'entre eux étant sous traitement ARV majoritairement de première ligne, associant deux inhibiteurs nucléosidiques de la transcriptase inverse (INTI) et un inhibiteur non nucléosidique de la transcriptase inverse (INNTI)). Le taux de CD4 moyen était de 108,7 cellules/mm^3^. Parmi les 36 patients, 30 d'entre eux avaient une infection opportuniste dont 12 cas de tuberculose, 15 cas d'une infection bactérienne sévère et deux cas de pathologie tumorale. Les antécédents de tuberculose ou autres pathologies (diabète, HTA) ont été rapportés chez 17 patients. La notion d'hospitalisation antérieure avant l'admission au SMIT pour une durée moyenne de 25±17 jours a été retrouvée chez 15 patients. Ces données sont résumées dans le Tableau 1.

**Tableau 1:** données générales des patients de l’étude, SMIT, 2005-2015, N = 36

Variables	Total (n=36)
Age (années), moyenne (ET)	43,3 (±12)
**Sexe n (%)**	
Féminin	23 (64)
Homme	13 (36)
**Profession n (%)**	
Oui	28 (78)
Non	08 (22)
**Type de VIH n (%)**	
VIH1	35 (97)
VIH2	01 (03)
**Durée de l’histoire VIH n(%)**	
Moins de 1 an	13 (36)
1-5 ans	13 (36)
>5 ans	10 (28)
**Antécédents n(%)**	
Tuberculose	09 (25)
Hospitalisation antérieure	15 (42)
Diabète ou HTA	08 (22)
**IO associés n (%)**	
Oui	30 (83)
Non	06 (17)
**Type d’IO n (%)**	
Tuberculose	12 (33)
Candidose orale	11 (31)
Toxoplasmose Cérébrale	03 (8,3)
Cryptococcose neuroméningée	01 (2,7)
Herpès génital	01 (2,7)
Pneumocystose	01 (2,7)
Mycobactériose atypique	01 (2.7)
**Comorbidités (n%)**	
Infection bactérienne sévère	15 (41,7)
Pathologie tumorale	02 (0,5)
Taux de CD4, Moyenne (±ET)	108,7 (±202,3)
**Traitement ARV n (%)**	
Oui	25 (69)
Non	11 (31)
**Si ARV régime n (%)**	
2INRT + INNRT	16 (64)
2 INRT+ IP/rit	08 (32)
2 INRT+ Anti intégrase	01 (04)

ET; Ecart-type, Stade CDC: Classification de l’infection à VIH/SIDA chez l’adulte; IO: Infection Opportuniste;

Traitement ARV: Traitement antirétroviral; INRT: Inhibiteur Nucléosidique de la Transcriptase Inverse, INNRT: Inhibiteur Non Nucléosidique de la Transcriptase Inverse

### Données relatives aux manifestations thromboemboliques

Chez les patients ayant présenté l'EP (n = 26), le diagnostic a été posé dès l'admission chez 5 (56%) patients. La symptomatologie clinique était dominée par le ballotement du mollet (89%), la toux (78%) et la fièvre (67%). On notait la présence d'une dyspnée chez 56% des patients. L'EP était localisée à droite dans 100% des cas. Les artères pulmonaires étaient concernées dans 7 cas au niveau du tronc principal, 8 cas au niveau des branches segmentaires et 7 cas au niveau des branches sous-segmentaires. La thrombose artérielle était bilatérale intéressant les carotides internes gauche et droite. Concernant les patients ayant présenté une TVP (n = 9), le signe fonctionnel prédominant était une grosse jambe douloureuse (73%) et le signe physique le plus retrouvé était le signe de Homans (85%). Concernant la répartition des TVP selon la veine atteinte, l'on constate que les lésions étaient majoritairement unilatérales (81%) et à gauche (77%) et concernaient les veines fémorales et poplitées (39% et 35%) majoritairement (Tableau 2). L'ensemble des patients a bénéficié d'un traitement anticoagulant à base d'enoxaparine majoritairement (89% des cas) ou de calciparine. Divers traitements ont été associés dans 92% des cas en fonction des pathologies associées à la maladie thromboembolique (Tableau 3). Il s'agissait notamment des antituberculeux prescrits chez 12 (33%) patients, des antibiotiques chez 24 (67%) patients. Sur le plan évolutif nous avons enregistré 09 (25%) décès dont 19% chez les patients qui ont présenté une EP et 5% chez les patients atteints de TVP.

**Tableau 2:** descriptif des manifestations thromboemboliques, SMIT, 2005-2015, N = 36

Manifestations thromboemboliques	Effectif	Pourcentage
Embolie pulmonaire *Clinique*	N = 9	
**Délai de diagnostic**		
A l’admission	05	56
Au-delà de 24h	04	44
Fièvre	06	67
Ballotement du mollet	08	89
Dyspnée	05	56
Douleur thoracique	02	22
Toux	07	78
***Topographie des lésions***		
Unilatérale	03	33
Bilatérale	06	67
Gauche	06	67
Droite	09	100
***Artères pulmonaires***		
Tronc principal	07	78
Branche segmentaires	08	89
Branches sous-segmentaires	07	78
Thrombose veineuse profonde		
*Clinique*	N=26	
**Délai de diagnostic**		
A l’admission	21	81
Grosse jambe douloureuse	19	73
Signe de Homans présent^a^	22	85
Ballotement du mollet	17	65
***Topographie des lésions***		
Unilatérale	21	81
Bilatérale	05	19
Gauche	20	77
Droite	06	23
***Veines atteintes***		
Fémorales	10	39
Poplitées	09	35
Iliaques	01	04
Surales	04	15
Autres	02	08
Thrombose artérielle *Clinique*	N=01	
**Délai de diagnostic**		
Au-delà de 24h	01	100
Fièvre	01	100
Ballotement du mollet	01	100
***Topographie des lésions***		
Bilatérale	01	100
*Artères atteintes*		
Carotides internes (droite et gauche)	01	100

a une douleur provoquée du mollet à la dorsiflexion du pied. C'est un signe recherché lors d'une suspicion de thrombose veineuse profonde à l'examen clinique, mais il ne peut pas faire le diagnostic; NB: 6 cas des embolies pulmonaires étaient associés à une thrombose veineuse profonde

**Tableau 3:** données thérapeutiques et évolutives des patients de l’étude, SMIT, 2005-2015, N = 36

Variables	Effectif	Pourcentage
Traitement de la thrombose		
Enoxaparine		
Oui	32	89
Non	04	11
Calciparine		
Oui	04	11
Non	32	89
AVK		
Oui	20	56
Non	16	44
Association HBPM-AVK		
Oui	06	17
Non	30	83
Autres traitements		
-Antituberculeux	12	33
-Antibiotiques	24	67
Décès		
Oui	09	25
Non	27	75

AVK: Antivitamine K; Association HBPM-AVK : Association Heparine de Bas Poids Moléculaire et Antivitamine K

## Discussion

Notre étude a permis de confirmer le risque de la MTE chez les patients infectés par le VIH à un stade avancé et ayant une pathologie opportuniste sévère associée. A ce jour, il est clairement établi que les patients infectés par le VIH ont un risque accru de développer une maladie thromboembolique comparés à la population générale non infectée par le VIH [15, 16]. En 2005, dans une revue systématique de la littérature, dix études épidémiologiques pertinentes ont examiné le risque de TVP chez les sujets infectés par le VIH avec des incidences variant entre 0,2 et 7,2% [17]. Egalement, l'une des plus grandes études américaines conduite dans plusieurs Etats et portant sur 42.935 patients avant l'ère des multi thérapies antirétrovirales, a rapporté une incidence à 2,6 pour 1.000 personnes/année [18]. Dans cette étude, les facteurs indépendamment associés à la thrombose veineuse étaient l'âge > 45 ans, un antécédent connu d'infection à cytomégalovirus ou de pathologies opportunistes en cours, l'utilisation d'antiprotéase dans la trithérapie ou un antécédent d'hospitalisation [18]. Notre étude non comparative ne portant que sur des cas rapportés ne nous permet pas d'identifier des facteurs associés à la MTE. Par contre, nous notons à l'instar des autres séries qu'un nombre important de nos patients était infecté par *Mycobacterium tuberculosis*, bactérie pouvant induire la synthèse d'Ac anticardiolipin, et la production de cytokines (TNF-α, IL-1, IL-6) à l'origine d'un état d'hypercoagulabilité [19].

Concernant le diagnostic topographique, la localisation prédominante de la TVP dans notre série était aux membres inférieurs, siège majoritairement retrouvée dans toutes les études sur la MTE chez les sujets infectés par le VIH, avec des chiffres variant de 45 à 75% [20-22]. Il existe un risque relatif de 3,9 entre l'infection à VIH et la TVP bilatérale des membres inférieurs (association significative) de même que la présence d'une thrombose iliaque ou fémorale [21]. Des localisations thrombotiques plus rares ont été décrites, notamment des thromboses de la veine porte et de la veine rénale. Deux cas de thrombose de localisation inhabituelle ou exceptionnelle chez des patients infectés par le VIH ont été décrits par Konin *et al.* Ces localisations concernaient la thrombose de la veine mésentérique supérieure et la thrombose cérébrale [23]. L'EP représentait 33% contre 6 cas sur 10 (60%) de TVP des membres inférieurs et 3 cas sur 10 (30%) d'EP dans l'étude de Saif *et al.* [24].

Le travail objective aussi le pronostic sévère de ces atteintes cardio-vasculaires avec près d'un tiers de décès constaté dans notre série, ce qui est plus élevé par rapport aux séries précédentes rapportées dans la littérature dans lesquelles la létalité variait entre 4% et 10% [25, 26]. Dans notre contexte, la comorbidité de nos patients pourrait expliquer ce taux élevé de décès. Ce travail rétrospectif présente quelques limites en rapport d'une part avec la faible taille de notre échantillon et l'absence de données dans les dossiers des patients de bio-marqueurs de l'hypercoagulabilité conférant l'état prothrombotique chez ces patients.

## Conclusion

L'infection à VIH/SIDA est à l'origine d'un état d'hypercoagulabilité. Le risque de MTE est assez élevé chez les VIH positifs surtout en présence des infections opportunistes. Notre étude souligne l'enjeu du respect des mesures prophylactiques antithrombotiques chez ces patients lorsqu'ils sont hospitalisés et la nécessité de l'élaboration d'algorithmes consensuels pour une prise en charge adéquate de ces patients. Enfin des études supplémentaires sont nécessaires pour confirmer l'association entre VIH et maladie thromboembolique.

### Etat des connaissances actuelles sur le sujet

- Manifestations cardiovasculaires, question majeure des maladies non transmissibles constituant une menace avec un risque plus élevé chez le sujet infecté par le VIH que dans la population générale;
- Maladies cardiovasculaires plus fréquentes avec les cancers et les pathologies bactériennes sévères chez le sujet infecté par le VIH;
- Incidence plus élevée des manifestations thromboemboliques dans les pays du Nord que dans les pays du Sud.

### Contribution de notre étude à la connaissance

- Données additionnelles sur la maladie thromboembolique en Afrique de l'Ouest et tout particulièrement en Côte d'Ivoire;
- Manifestations thromboemboliques généralement sévères engageant le pronostic vital chez les patients infectés par le VIH;
- Faire des recommandations sur un modèle différencié de soins dans les maladies thromboemboliques.

## Conflits d’intérêts

Les auteurs ne déclarent aucun conflit d’intérêts.
